# Endosomal Rab GTPases regulate secretory granule maturation in *Drosophila* larval salivary glands

**DOI:** 10.1080/19420889.2021.1874663

**Published:** 2021-02-09

**Authors:** Cheng-I Jonathan Ma, Julie A. Brill

**Affiliations:** a Cell Biology Program, The Hospital for Sick Children, Toronto, ON, Canada; b Institute of Medical Science, University of Toronto, Medical Sciences Building, Toronto, ON, Canada; c Department of Molecular Genetics, University of Toronto, Medical Sciences Building, Toronto, ON, Canada

**Keywords:** Rab GTPase, trafficking, secretory granule, salivary gland, Drosophila

## Abstract

Secretory granules (SGs) are organelles responsible for regulated exocytosis of biologically active molecules in professional secretory cells. Maturation of SGs is a crucial process in which cargoes of SGs are processed and activated, allowing them to exert their function upon secretion. Nonetheless, the intracellular trafficking pathways required for SG maturation are not well defined. We recently performed an RNA interference (RNAi) screen in *Drosophila* larval salivary glands to identify trafficking components needed for SG maturation. From the screen, we identified several Rab GTPases (Rabs) that affect SG maturation. Expression of constitutively active (CA) and dominant-negative (DN) forms narrowed down the Rabs important for this process to Rab5, Rab9 and Rab11. However, none of these Rabs localizes to the limiting membrane of SGs. In contrast, examination of endogenously YFP-tagged Rabs (YRabs) in larval salivary glands revealed that YRab1 and YRab6 localize to the limiting membrane of immature SGs (iSGs) and SGs. These findings provide new insights into how Rab GTPases contribute to the process of SG maturation.

## Introduction

Regulated secretion is a fundamentally important process in animal physiology. SGs are organelles responsible for storage, processing, and release of biologically active molecules that regulate homeostasis. Biogenesis of SGs starts at the endoplasmic reticulum (ER), where SG cargo proteins are synthesized. These cargo proteins are then trafficked through the Golgi and concentrated at the *trans-*Golgi network (TGN). iSGs bud from the TGN through cargo aggregation and the help of coat and adapter proteins. Maturation of iSGs takes place through homotypic fusion and remodeling, which ensure proper packaging and processing of cargoes as well as removal of unneeded material. Thus, proper maturation of iSGs is required for the activity of cargo proteins following exocytosis. However, the intracellular trafficking machinery required for SG maturation is not well understood.

The larval salivary glands of *Drosophila melanogaster* are a highly accessible genetic model system for studying SG biogenesis [1–4]. Salivary glands begin production of adhesive mucin-like glue proteins 24 h after entering the third instar larval (L3) stage. The glue proteins are packaged into SGs known as glue granules. These SGs mature over the next 18 h and are released in response to a pulse of the steroid hormone ecdysone to adhere the larvae onto a solid surface for pupariation and metamorphosis [1,2]. Mature SGs are 2- to 4-fold larger in cross-sectional surface area than iSGs, allowing a visual screen for genes required for proper SG maturation using the fluorescently tagged cargo protein Sgs3.

## Materials and methods

### Fly genetics

Flies were raised on standard cornmeal molasses agar at 25°C [5]. The following stocks were acquired from Bloomington *Drosophila* Stock Center (BDSC; stock numbers are listed): UAS-*Rab5* RNAi (#34832, *P{TRiP.JF03335}attP2*); UAS-*Rab6* RNAi (#27490, *P{TRiP.JF02640}attP2*); UAS-*Rab11* RNAi #1 (#27730, *P{TRiP.JF02812}attP2*); UAS-*Rab11* RNAi #2 (#42709, *P{UAS-Rab11.dsRNA.WIZ}F3-B*), UAS-*RabX6* RNAi (#26281, *P{TRiP.JF02050}attP2*); UAS-YFP-Rab5^CA^ (#9774, *P{UASp-YFP.Rab5.Q88L}*); UAS-YFP-Rab5^DN^ (#9771, *P{UASp-YFP.Rab5.S43N}*); UAS-YFP-Rab6^CA^ (#9776, *P{UASp-YFP.Rab6.Q71L}*); UAS-YFP-Rab6^DN^ (#23249, *P{UASp-YFP.Rab6.T26N}*); UAS-YFP-Rab9^CA^ (#9785, *P{UASp-YFP.Rab9.Q71L}*); UAS-YFP-Rab9^DN^ (#23643, *P{UASp-YFP.Rab9.S26N}*); UAS-YFP-Rab11^CA^ (#9791, *P{UASp-YFP.Rab11.Q70L}*); UAS-YFP-Rab11^DN^ (#9792, *P{UASp-YFP.Rab11.S25N}*); UAS-YFP-Rab32^CA^ (#9816, *P{UASp-YFP.Rab32.Q79L}*); UAS-YFP-Rab32^DN^ (#23281, *P{UASp-YFP.Rab32.T33N}*); UAS-YFP-RabX6^CA^ (#23646, *P{UASp-YFP.RabX6.M69L}*); UAS-YFP-RabX6^DN^ (#9856, *P{UASp-YFP.Rab6.S22N}*); AB1-GAL4 (#1824, *P{GawB}AB1-GAL4*). Endogenously tagged YRab lines from BDSC included YRab1 (#62539 {*TI{TI}Rab1^EYFP^*}); YRab6 (#62544, {*TI{TI}Rab6^EYFP^*}); and additional stocks from the BDSC YRab collection (https://bdsc.indiana.edu/stocks/gfp/rab_eyfp.html). Sgs3-DsRed under control of the *Sgs3* promoter (*P{w^+^, Sgs3-DsRed}*) was a gift from A. Andres (Costantino et al., [6]; University of Nevada, Las Vegas, NV, USA). All UAS lines were expressed in salivary gland cells under control of the AB1-GAL4 driver.

### Live microscopy

Salivary glands were dissected from late L3 larvae in 50 µL *Drosophila* Ringer’s solution (10 mM Tris, 182 mM KCl, 46 mM NaCl, 3 mM CaCl_2_ · 2H_2_O, PH 7.2) using fine forceps. Dissected salivary glands were mounted in 8 µL *Drosophila* Ringer’s solution using self-adhesive reinforcement labels (Avery #32203, USA) as spacers and sealed with nail polish. Samples were imaged using a Quorum spinning disc confocal coupled with an Olympus IX81 microscope (Quorum Technologies Inc., Canada). Images were acquired using a 60X oil objective (NA 1.4) and Volocity 6.3 (PerkinElmer, USA) software. Serial optical sections were acquired at an interval of 0.3 µm for a total of 20–30 µm. Images were adjusted for brightness and contrast using Adobe Photoshop Creative Cloud (Adobe, USA).

## Results

**Figure 1.**
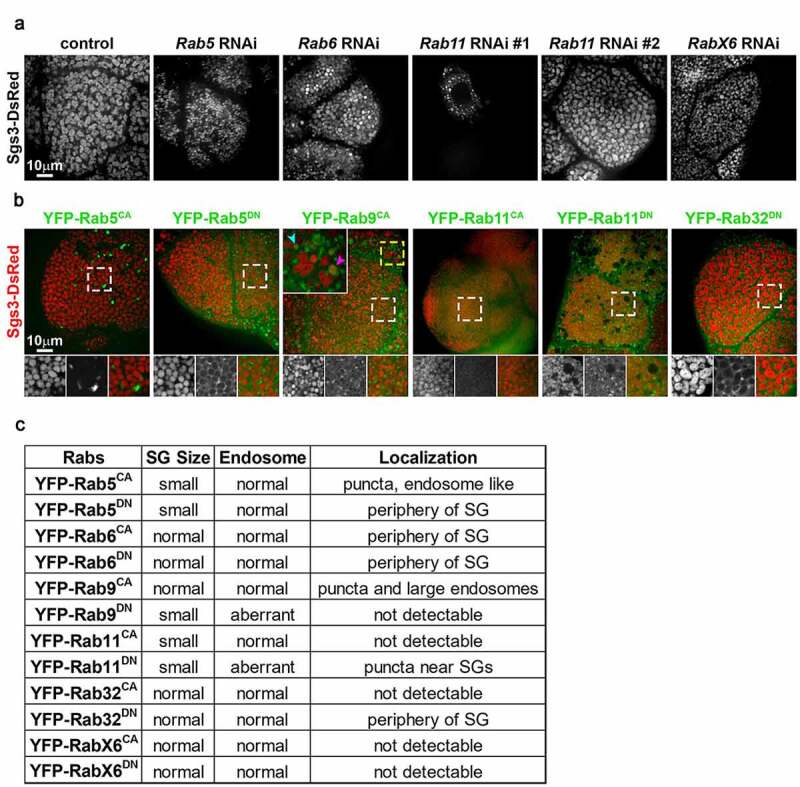
Rab GTPases needed for SG maturation

We previously performed a candidate RNAi screen to identify trafficking genes that regulate SG maturation [7]. Transgenic RNAi lines expressing short hairpin RNAs targeting each of the *Drosophila* Rabs were included in the screen, and we identified several Rab GTPases that are required for normal SG maturation. RNAi lines targeting *Rab5, Rab6, Rab11* or *RabX6* resulted in SGs of reduced size when compared to controls (Figure 1a). To confirm that these Rabs play roles in SG maturation and to examine their localization, we overexpressed YFP-tagged CA and DN forms of these Rabs in larval salivary glands (Figure 1b,C). In addition to Rab5, Rab6, Rab11 and RabX6, we also overexpressed CA and DN forms of Rab9 and Rab32. Rab9 was chosen because our previous results had suggested that retrograde trafficking from late endosomes (LEs) might be important for SG maturation [3]. Rab32 was evaluated because we suspected that SGs in the larval salivary glands might share trafficking machinery with lysosome-related organelles [8]. Overexpression of Rab5^CA^, Rab5^DN^, and Rab11^CA^ all resulted in smaller SGs, indicating impaired SG maturation. Rab5^CA^ localized to puncta resembling endosomes, whereas Rab5^DN^ weakly localized around SGs and concentrated strongly in crescents around SGs. Although overexpression of Rab11^CA^ reduced SG size, Rab11^CA^ fluorescence was too weak to reveal its localization. Overexpression of Rab9^CA^ and Rab11^DN^ also disrupted SG maturation, as SGs were reduced in size. In Rab^CA^ overexpressing cells, there were small (cyan arrowhead, large inset) and large (magenta arrowhead, large inset) round compartments that had weak Sgs3-DsRed signal and were often labeled by Rab9^CA^. Rab11^DN^ overexpressing cells also exhibited small and large compartments containing weak or no Sgs3-DsRed, but Rab11^DN^ was absent from the large compartments, suggesting these might be aberrant LEs. Although overexpression of Rab32^DN^ had little effect on SG maturation, Rab32^DN^ decorated SGs in a manner similar to Rab5^DN^.

(A-B) Spinning-disc confocal images of live L3 salivary gland cells. (A) Cells expressing the glue granule marker Sgs3-DsRed (gray) in different RNAi backgrounds. (B) Cells expressing Sgs3-DsRed (red) with YFP fusions to CA or DN forms of different Rabs (green). Region marked by yellow dashed box (magnified two-fold in large inset) shows examples of small and large YFP-Rab9^CA^-postive compartments containing low levels of Sgs3-DsRed (labeled by cyan and magenta arrowheads, respectively). Regions marked by white dashed boxes are magnified 1.67-fold in insets. (C) Table summarizing phenotypes observed for larval salivary gland cells expressing CA or DN forms of different YFP-Rabs.

**Figure 2.**
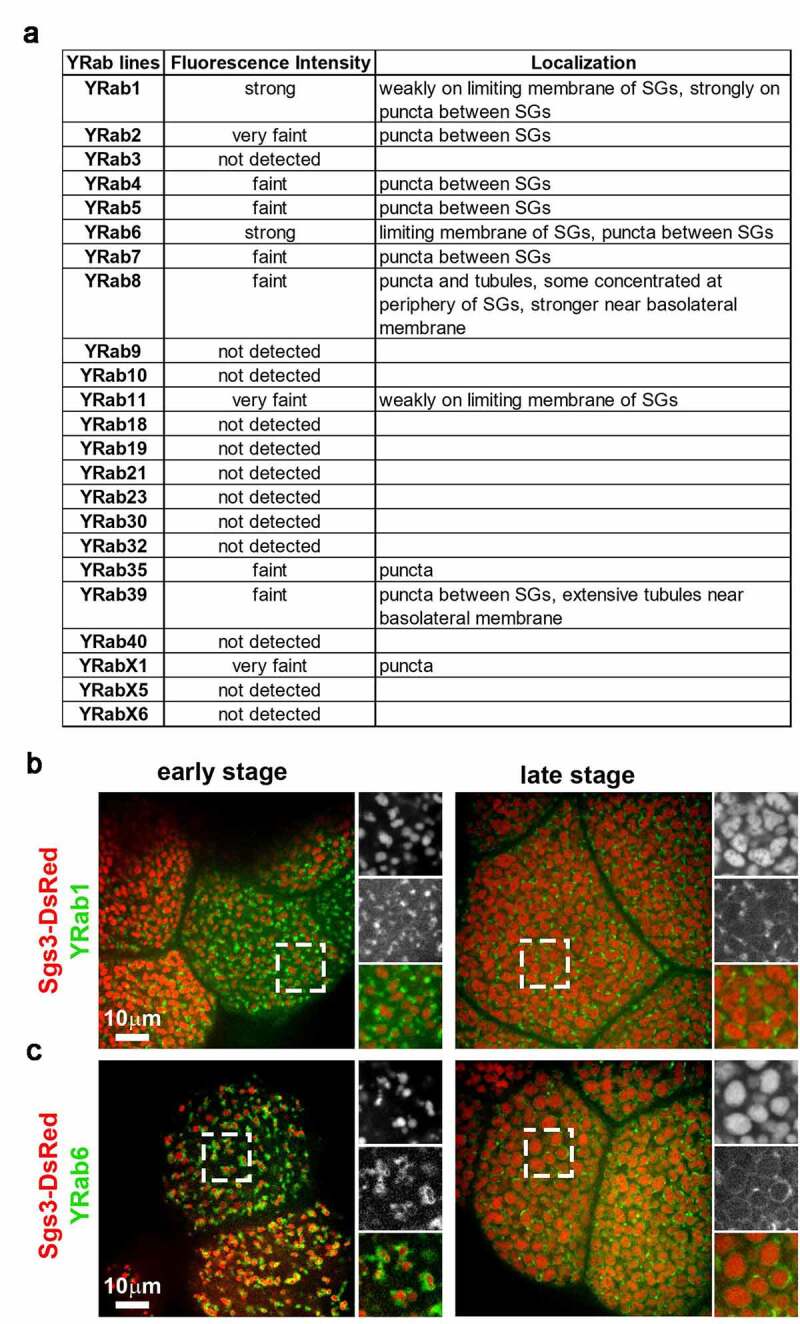
Rab1 and Rab6 decorate limited membranes of SGs

To evaluate the localization of Rab GTPases without overexpression, we examined 23 out of 27 lines expressing endogenously YFP-tagged Rabs (YRabs) [9]. The other four lines (YRab14, YRab26, YRab27, YRabX4) were omitted because salivary gland expression of these YRabs was undetectable by immunoblotting and fluorescence microscopy [9]. Most of the YRabs did not localize to SGs or had low expression levels (Figure 2a). The fluorescence intensity of many YRabs was lower than previously reported [9] because we had to cross the YRab lines with flies expressing Sgs3-DsRed, and the resulting offspring contained only one copy of the YRab allele. Out of the 23 YRabs tested, YRab1 and YRab6 localized to SGs. YRab1 localized to the limiting membrane of iSGs and SGs during early and late stages of SG development, respectively (Figure 2b). YRab6 also localized to the limiting membrane of iSGs and SGs (Figure 2c). In addition, YRab6 appeared to localize more strongly to SG membranes than YRab1.

(A) Table summarizing fluorescence intensity and distribution of different YRabs in relation to Sgs3-DsRed. (B-C) Spinning-disc confocal images of live L3 salivary gland cells expressing Sgs3-DsRed (red) together with endogenously tagged YRabs. (B) Early (left) or late (right) stage salivary gland cells expressing YRab1 (green). (C) Early (left) or late (right) stage salivary gland cells expressing YRab6. Marked regions are magnified 1.67-fold in insets.

## Discussion

Rabs regulate intracellular trafficking between organelles and are important for maintaining organelle identity [10]. Several Rabs are necessary for normal SG function in cultured cell lines derived from professional secretory cells. Rab3 and Rab27 have roles in tethering and docking of SGs at the PM for exocytosis [11–17]. Other Rab GTPases involved in this process include Rab11 and Rab37, but their roles are not as well defined [18–23]. In this study, we identified Rabs that associate with SGs and those that regulate SG maturation in the *Drosophila* larval salivary gland. Based on our results, it appears that Rab5, Rab6, Rab9 and Rab11 play important roles in SG maturation.

From our RNAi experiments, cells expressing Rab5, Rab6, and Rab11 RNAi not only exhibited small SGs but also appeared to have smaller cell size when compared to controls. Rab5, Rab6, and Rab11 are important for intracellular trafficking and a decrease in their expression level could affect signaling of receptors involved in cell growth [24,25]. Moreover, these Rabs can also regulate autophagy and thus affect cell growth and size when knocked down [26–30].

Rab5 is required for normal SG maturation and size in mast cells [31]. In addition, depleting Rab5 with RNAi inhibits SNAP23-mediated homotypic fusion of SGs during compound secretion [32]. Our previous study suggested that Rab5-dependent early endosome (EE) sorting is needed for SG maturation [7]. Here, we demonstrate that overexpressing Rab5^CA^ or Rab5^DN^ impaired SG maturation and that Rab5^CA^ localized to puncta resembling EEs, whereas Rab5^DN^ localized to the periphery of SGs. This further confirms Rab5-mediated sorting is important for normal SG maturation and suggests that Rab5 may cycle between EE and SG when it is GTP-bound or GDP-bound. Although YFP-Rab32^DN^ localized to the periphery of SGs in a manner similar to YFP-Rab5^DN^, endogenous Rab32 has low transcript expression [33,34], and YRab32 protein expression is not detectable in larval salivary glands [9]. Thus, Rab32 is unlikely to participate in SG biogenesis in this tissue.

Although Rab6 RNAi led to a defect in SG maturation, expression of Rab6^CA^ and Rab6^DN^ had no effect. According to both our data on YRab6 and the original characterization of YRab lines by Dunst et al., *Drosophila* larval salivary glands have a very strong expression of Rab6 [9]. It is likely that expression of Rab6^CA^ and Rab6^DN^ was not high enough to outcompete endogenous Rab6 and generate a phenotype.

Expression of Rab9^DN^, Rab11^CA^ or Rab11^DN^ inhibited SG maturation. Although these Rabs do not have clear association with SGs, impairing Rab9-mediated LE to Golgi retrograde trafficking [35] or Rab11-mediated recycling endosome to Golgi retrograde trafficking [36,37] is likely detrimental for this process. Together with the data on Rab5, this suggests that multiple retrograde pathways are needed for normal SG maturation. Our previous characterization of *PI4KII* null mutants, which show defects in SG maturation, further supports this observation, as both EE and LE sorting defects were observed [3,7]. Moreover, our genetic screen identified multiple retrograde trafficking factors, including subunits of Golgi-associated retrograde protein complex, Past1/EHD1, Arl1, Snx3, Vps13, Lqfr/Epsin-2, and Syx16 [7].

Examination of endogenously tagged YRabs in the salivary gland revealed that YRab1 and YRab6 are associated with SGs. Both Rab1 and Rab6 localize to the Golgi. Rab1 is needed for ER to Golgi trafficking, whereas Rab6 is important for trafficking between Golgi cisternae, Golgi and TGN, and endosomes and TGN [38–40]. Our observation that Rab1 localizes to SGs is novel, as Rab1 has previously been shown to localize to the Golgi and to ER exit sites in *Drosophila* [41,42]. On the other hand, our observations are consistent with studies showing the association of Rab6 with secretory granules in other systems. For example, Rab6 associates with zymogen granules from pancreas, atrial granules from atrial myocytes, and SGs from *Toxoplasma gondii* [43–45]. *Rab1* RNAi did not disrupt SG maturation, but this could be the result of inefficient knockdown. On the other hand, Rab6 is clearly involved in SG maturation. Because Rab6 has multiple roles in intracellular trafficking, additional experiments are needed to clarify how Rab6 contributes to this process.

In conclusion, our results provide evidence that multiple Rab-dependent retrograde trafficking pathways from endosomal compartments are required for normal SG maturation, as knockdowns of Rab5, Rab9 and Rab11 disrupted this process. Furthermore, Golgi-localized Rab1 and Rab6 associate with the limiting membrane of SGs, and Rab6 might also contribute to trafficking between endosomes and TGN. In the future, it will be of interest to uncover the mechanism by which these Rabs regulate SG maturation in larval salivary glands and other systems.
